# Refining treatment strategies for non-small cell lung cancer lacking actionable mutations: insights from multi-omics studies

**DOI:** 10.1038/s41416-025-03139-6

**Published:** 2025-08-23

**Authors:** Andrea Rocca, Lucio Crinò, Luca Braga, Francesco Salton, Barbara Ruaro, Marco Confalonieri, Daniele Generali, Paola Confalonieri

**Affiliations:** 1 Department of Medical, Surgical and Health Sciences, University of Trieste, Trieste, Italy; 2 IRCCS Istituto Romagnolo per lo Studio dei Tumori (IRST) ‘Dino Amadori’, Meldola, Italy; 3 Functional Cell Biology Laboratory, International Centre for Genetic Engineering and Biotechnology, Trieste, Italy; 4 Pulmonology Unit, Azienda Sanitaria Universitaria Giuliano Isontina (ASUGI), Trieste, Italy; 5 Breast and Brain Unit, ASST Cremona, Cremona, Italy

**Keywords:** Non-small-cell lung cancer, Cancer genomics, Transcriptomics, Proteomics, Target identification

## Abstract

Non-small cell lung cancer (NSCLC) represents a heterogeneous group of malignancies characterised by diverse histological and molecular features. Some NSCLCs, particularly adenocarcinomas, harbour genomic alterations in receptor tyrosine kinases or downstream RAS/RAF signalling pathways, which are targets of effective therapies. NSCLCs lacking actionable genomic alterations often benefit from immune checkpoint inhibitors, though only a minority of patients achieve long-term survival. These tumours often carry alterations in tumour suppressor genes like *TP53*, *KEAP1*, *STK11*, or *NF1*, for which pharmacological strategies are still under investigation. This review explores emerging therapeutic opportunities unveiled by multi-omics studies in NSCLCs without actionable genomic alterations. Proteogenomic approaches—integrating genomic, transcriptomic and proteomic data—enable a comprehensive understanding of NSCLC molecular landscapes and signalling network dysregulation, helping to identify distinct tumour subtypes and potential therapeutic targets. These tumours exhibit alterations in cell cycle regulation, DNA repair, immune signalling, epigenetic modulation and metabolic and redox pathways. Although therapies targeting tumour suppressor genes like p53 remain highly anticipated, extending our understanding of the broader molecular landscape in these tumours may reveal novel vulnerabilities and inform the development of novel drugs or combination strategies. This could further advance precision oncology for NSCLC.

## Introduction

Non-small-cell lung cancer (NSCLC) is the leading cause of cancer-related death worldwide [1]. It encompasses several histological types, including adenocarcinoma (LUAD), squamous cell carcinoma (LUSC), large cell carcinoma and rarer types [2]. NSCLC has a high rate of somatic mutations [3], contributing to its biological complexity and treatment resistance.

With advancements in understanding 'oncogene addiction', molecular profiling has become essential for identifying actionable genetic alterations [4–8]. These include several types of *EGFR* mutations (present in 10–20% of Caucasian populations and up to 50–60% of Asian populations [9, 10]), rearrangements involving *ALK*, *ROS1*, *RET*, *NTRK*, or NRG1, *MET* exon 14 skipping mutations and *BRAFV600E*, *KRASG12C*, or *HER2* mutations. Other driver alterations under clinical investigation are *HER2* and *MET* amplifications. LUAD exhibits a higher prevalence of actionable genomic alterations compared to other histological types, and targeted therapies often represent the first-line treatment when these alterations are detected [11].

Tumours with actionable driver alterations represent about 25–30% of NSCLC cases and up to 60% of LUAD [12]. For tumours lacking such mutations, treatment is typically guided by the expression of Programmed Death-Ligand 1 (PD-L1), a marker predictive of response to immune checkpoint inhibitors (ICIs). Despite the advancements in immunotherapy, many patients do not respond or eventually progress due to primary or secondary resistance. Long-term response rates in the range of 20% after first-line immunotherapy alone [13] and 20–30% after chemo-immunotherapy [14, 15] highlight the need to develop additional treatments.

The genomic characterisation of NSCLC has been pivotal in identifying therapeutic targets, which have been validated by the clinical efficacy of matched targeted therapies, paving the way for precision oncology [16]. Nonetheless, this approach has notable limitations. Certain genetic alterations remain undruggable, and even when a targetable mutation is present, the corresponding therapy does not always yield a clinical benefit. Additionally, not all genomic changes result in phenotypic consequences, and other molecular mechanisms may play critical roles in tumour development and progression [17–19].

In recent years, substantial advances in omics technologies, including transcriptomics, proteomics, phosphoproteomics, metabolomics, epigenomics and others, have expanded our capacity to investigate the molecular complexity of cancer [20]. By analyzing multiple molecular layers, these approaches offer complementary perspectives, and their integrative application can allow a more comprehensive characterisation of the biomolecular alterations driving tumorigenesis and the identification of novel therapeutic targets [17, 19, 21].

This review article examines the molecular alterations discovered through multi-omics studies in NSCLC, with an emphasis on proteogenomic studies, focusing on tumours lacking known actionable genomic alterations and highlights the potential of multi-omics approaches as research tools to identify new therapeutic targets. Data on tumours with actionable genomic alterations are reported only when they pertain to potential targets not yet utilised in clinical practice. We then provide a brief overview of emerging therapeutic strategies designed to target alterations in tumour suppressor genes and conclude by discussing the challenges associated with integrating multi-omics data, while proposing a potential roadmap for their clinical validation.

A systematic literature search was conducted on Pubmed up to April 2025, using various combinations of the following terms, both as free-text and MESH terms: 'Lung Neoplasms', 'Carcinoma, Non-Small-Cell Lung', 'Adenocarcinoma of Lung', 'Carcinoma, Squamous Cell', 'Carcinoma, Large Cell', 'Genomics', 'Transcriptome', 'Proteogenomics', phosphoproteomic (not MESH). Additional material was sought on Scopus, Google Scholar and through manual review of reference lists from relevant articles. The review focused on studies containing original omics data. Articles limited to bioinformatic analyses from public databases, or addressing individual or small sets of genes, or focused specifically on response or resistance to existing therapies, were excluded.

## NSCLC genomic profiling

Comprehensive genomic profiling using next-generation sequencing, entailing either a wide targeted gene panel or whole exome sequencing (WES) or whole genome sequencing (WGS), has uncovered a variety of somatic genomic alterations in NSCLC [22–27]. They show significant differences not only among histological types [9, 28] but also according to ethnicity [9, 10, 29], gender [30] and smoking history [25]. It is important to note that the criteria for defining significantly mutated genes can vary across studies, complicating direct comparisons. Furthermore, identifying cancer-related genes is particularly challenging in tumours with a high tumour mutational burden (TMB), where the majority of genomic alterations are passengers rather than drivers.

### Genomic profiling of lung adenocarcinoma

In primary LUAD, driver mutations interest both oncogenes, many of which have clinical relevance being druggable, and tumour suppressor genes [23–25, 27, 28]. Among the first are mutations of receptor tyrosine kinases (RTK) [31] from families like *ERBB/EGFR*, *FGFR* and *MET*. Other RTKs, such as *ALK*, *RET*, *ROS1* and *NTRK*, are altered by translocations. Further driver oncogenes belong to the downstream signalling pathways and include *KRAS*, *BRAF* and *PIK3CA*. Altered tumour suppressor genes include *TP53*, *KEAP1*, *STK11* and *NF1*, among others. Mutations also occur in chromatin-modifying genes, such as *ARID1A*, *ARID2*, *SETD2*, *SMARCA4* and *MLL3* (*KMT2C*), leading to epigenomic abnormalities that overlap with genomic alterations, further complicating lung cancer biology. Table 1 provides a list of genes significantly mutated in NSCLCs, described in the work of Campbell et al. [28] and found in OncoKB^TM^ [32, 33], along with potential therapeutic implications reported by OncoKB^TM^. OncoKB^TM^ provides the levels of evidence for altered genes as biomarkers predicting responsiveness to specific drugs in one or more types of neoplasm; however, this does not necessarily imply that the drug is recommended for clinical use in the specific context being considered. Genes encoding proteins already actionable in clinical practice are not shown. Table 2 provides a list of translocations found in NSCLC, reported by Campbell et al. [28].

**Table 1:** Significantly mutated genes in lung adenocarcinoma, lung squamous cell carcinoma and lung adenocarcinoma without actionable mutations and potentially active targeted drugs.

Cancer gene	OncoKB^TM^ gene type	Function or pathway	% Mut. LUAD	% Mut. LUAD wo	% Mut. LUSC	Drug	Drug class	Mechanism of action	OncoKB^TM^ leveled tumour type	OncoKB^TM^ highest level of evidence	OncoKB^TM^ level of evidence in NSCLC
*APC*	TSG	WNT	5			none					
*ARHGAP35*	Onc / TSG	GAP negatively regulating Rho-family small GTPases		7	6	none					
*ARHGEF12*	Onc / TSG	Rho GEF	4			none					
*ARID1A*	TSG	SWI/SNF chromatin-remodelling complex	7		7	PLX2853	small molecule	BRD4-inhibitor	All solid tumours	4	4
						tazemetostat	small molecule	EZH2 inhibitor	All solid tumours	4	4
*ARID2*	TSG	SWI/SNF chromatin-remodelling complex	5			none					
*ATM*^*b*^	TSG	DNA damage response	9			Olaparib	small molecule	PARP inhibitor	Prostate cancer	1	3B
						Talazoparib (+enzalutamide)	small molecules	PARP inhibitor + AR inhibitor	Prostate cancer	1	3B
*CDKN2A*	TSG	cell cycle	4		16	abemaciclib, palbociclib, ribociclib	small molecule	CDK4/6 inhibitors	All solid tumours	4	4
*CMTR2 (FTSJD1)*	TSG	RNA cap methyltransferase	4			none					
*CREBBP*	TSG	transcriptional co-activator with HAT activity		9		none					
*CTNNB1 (β-catenin)*	Onc	WNT	4			none					
*CUL3*	TSG	KEAP1/CUL3/NFE2L2 (oxidative stress response)			5	none					
*DOT1L*	Onc	histone H3K79 methyltransferase	3			none					
*FANCM*	TSG	DNA repair (homologous recombination)	7	12		none					
*FAT1*	TSG	cadherin superfamily member			14	none					
*FBXW7*	TSG	protein degradation			5	lunresertib + camonsertib	small molecule	PKMYT1 inhibitor + ATR inhibitor	Ovarian and endometrial cancer	3A	4
									All solid tumours	4	
*HRAS*	Onc	RAS/RAF/MEK/ERK			2	tipifarnib	small molecule	farnesyl protein transferase inhibitor	Urothelial carcinoma, HNSCC	3A	3B
*KARS*	?	aminoacyl-tRNA synthetases	2	4		none					
*KDM6A*	TSG	histone demethylase			5	tazemetostat	small molecule	EZH2 inhibitor	Bladder cancer	4	/
*KEAP1*	TSG	KEAP1/CUL3/NFE2L2 (oxidative stress response)	17	24		none					
*KMT2C (MLL3)*	TSG	histone methyltransferase	16	21		none					
*KMT2D (MLL2)*	TSG	histone methyltransferase			24	none					
*LATS1*	TSG	hippo		7		none					
*MAP2K1 (MEK1)*	Onc	RAS/RAF/MEK/ERK	2			cobimetinib, trametinib	small molecule	MEK1/2 inhibitor	LCH and other rare diseases	2	3A
									Melanoma, NSCLC, low-grade serous ovarian cancer	3A	
*MGA*	TSG	transcription factor involved in cell proliferation	6			none					
*NF1*	TSG	RAS/RAF/MEK/ERK	11	21	11	selumetinib, mirdametinib	small molecule	MEK1/2 inhibitor	neurofibroma	1	3B
						cobimetinib, trametinib	small molecule	MEK1/2 inhibitor	All solid tumours	4	4
*NFE2L2*	Onc	KEAP1/CUL3/NFE2L2 (oxidative stress response)			14	none					
*NOTCH1*	Onc/TSG	NOTCH			8	none for l.o.f. mutations found in LUSC					
*NRAS*^*a*^	Onc	RAS/RAF/MEK/ERK	1			cobimetinib, trametinib	small molecule	MEK1/2 inhibitor	LCH and other rare diseases	2	/
						binimetinib	small molecule	MEK1/2 inhibitor	melanoma	3A	3B
						binimetinib + ribociclib	small molecules	MEK1/2 inhibitor + CDK4/6 inhibitor	melanoma	4	/
*NSD1*	Onc / TSG	nuclear androgen receptor coregulator			6	none					
*PIK3CA*^a^	Onc	PI3K/AKT/mTOR	6		11	RLY-2608	small molecule	allosteric inhibitor of PI3Kα	All solid tumours	4	4
						alpelisib (+fulvestrant)	small molecules	PI3Kα inhibitor (+SERD)	Breast cancer	1	3B
						capivasertib (+fulvestrant)	small molecules	ATP-competitive pan-AKT inhibitor (+SERD)	Breast cancer	1	3B
						inavolisib + palbociclib (+fulvestrant)	small molecules	PI3Kα inhibitor + CDK4/6 inhibitor (+SERD)	Breast cancer	1	3B
*PTEN*	TSG	PI3K/AKT/mTOR			12	capivasertib (+fulvestrant)	small molecules	ATP-competitive pan-AKT inhibitor (+SERD)	Breast cancer	1	3B
						AZD8186, GSK2636771	small molecules	ATP-competitive PI3Kβ inhibitors	All solid tumours	4	4
*RAF1*	Onc	RAS/RAF/MEK/ERK	1			cobimetinib, trametinib	small molecule	MEK1/2 inhibitor	LCH and other rare diseases	2	/
									Histiocytosis	3A	
*RASA1*	TSG	GAP and negative regulator of RAS		5	6	none					
*RB1*	TSG	cell cycle	6		7	none					
*RBM10*	TSG	mRNA alternative splicing	6			none					
*RIT1*	Onc	small GTPase activating p38/MAPK	2			none					
*SETD2*	TSG	chromatin modulating (H3K36 trimethylase)	6			none					
*SMAD4*	TSG	TGF-ß	4			none					
*SMARCA4*	TSG	chromatin remodelling	9	14		PRT3789	small molecule	SMARCA2 degrader	NSCLC, oesophageal adenocarcinoma	3A	3A
*SOS1*	Onc	RAS/RAF/MEK/ERK		6		none					
*STK11*	TSG	AMPK/mTOR	16			bemcentinib + pembrolizumab	small molecule, monoclonal antibody	inhibitor of AXL RTK and anti-PD-1 antibody	NSCLC	4	4
*TP53*	TSG	p53	54	67	86	rezatapopt (PC14586)	small molecule	structural corrector specific for the Y220C-mutant p53	All solid tumours	3A	3A
*U2AF1*	Onc	splicing factor	3			emavusertib	small molecule	IRAK4 inhibitor	Acute myeloid leukaemia	4	/
						ceralasertib	small molecule	ATR inhibitor	CMML, myelodysplastic syndromes	4	/
*VAV1*	Onc	GEF		5		none					

Genes encoding proteins already actionable in clinical practice are not shown. Mutation data are taken from Campbell et al. [28]; targeted drug response predictions are taken from OncoKB^TM^ [32, 33] (last accessed on May 31st 2025). The level of evidence of the altered gene as biomarker predicting responsiveness to a drug is reported for the neoplasms with highest evidence and for NSCLC.

*AR* androgen receptor, *BRD*4 bromodomain-containing protein 4, *CDK*4/6 cyclin-dependent kinases 4 and 6, *CMML* chronic myelomonocytic leukaemia, *EZH*2, enhancer of zeste homologue 2, *GAP*, GTPase activating protein, *GEF* guanine nucleotide exchange factor, *HAT* histone acetyltransferase, *HNSCC* head and neck squamous cell carcinoma, *IRAK*4 interleukin-1 receptor-associated kinase 4, *IRF* interferon regulatory transcription factor, *LCH* Langerhans Cell Histiocytosis, *l*.*o*.*f*. loss of function, *LUAD* lung adenocarcinoma, *LUADwo* lung adenocarcinoma without actionable driver alterations, *LUSC* lung squamous cell carcinoma, *NSCLC* non-small cell lung cancer, *Onc* oncogene, *PARP* poly (ADP-ribose) polymerase, *RTK* receptor tyrosine kinase, *SERD* selective oestrogen receptor degraded, *SWI*/*SNF* SWItch/Sucrose Non-Fermentable, *TSG* tumour suppressor gene.

^a^Associations with some drugs and tumour types have been omitted because they were not relevant for NSCLC.

^b^PARP inhibitors are registered for prostate cancer in the presence of mutations in any of the homologous recombination genes; subgroup analysis is not significant for most individual mutations, including ATM mutations [187, 188].

Levels of evidence (OncoKB):

1. FDA-recognised biomarker predictive of response to an FDA-approved drug in this indication.

2. Standard care biomarker recommended by the NCCN or other professional guidelines, predictive of response to an FDA-approved drug in this indication.

3A. Compelling clinical evidence supports the biomarker as being predictive of response to a drug in this indication.

3B. Standard care or investigational biomarker predictive of response to an FDA-approved or investigational drug in another indication.

4. Compelling biological evidence supports the biomarker as being predictive of response to a drug. Most, but not all, associations in a specified cancer type that are OncoKB Levels 1, 2 or 3 A will propagate as Level 3B in other cancer types; level 4 alterations do not propagate to other indications (https://www.oncokb.org/faq).

**Table 2:** Fusion genes in lung adenocarcinoma and lung squamous cell carcinoma listed by frequency (only fusions found in ≥5 tumour samples are reported).

Lung adenocarcinoma (*N* = 2640)			Drug	Drug class	Mechanism of action	OncoKB^TM^ leveled tumour type	OncoKB^TM^ highest level of evidence	OncoKB^TM^ level of evidence in NSCLC
Fusions	*N* (%)	Kinases						
* C10orf68-CCDC7*	11 (0.4%)	no						
* CRHR1-KIAA1267*	11 (0.4%)	no						
* DHX40-RNFT1*	14 (0.5%)	no						
* EML4-ALK*	5 (0.2%)	ALK	alectinib, brigatinib, ceritinib, crizotinib, lorlatinib, ensartinib	Small molecule	ALK-inhibitors (some are multi-TKIs)	NSCLC	1	1
			NVL-655	Small molecule	ALK-inhibitor	NSCLC	3A	3A
* MYH9-COL1A1*	6 (0.2%)	no						
* PSAP-SQSTM1*	5 (0.2%)	no						
* RPS6KB1-VMP1*	12 (0.5%)	RPS6KB1						
* SFTPB-ANXA1*	5 (0.2%)	no						
* SFTPB-COL1A2*	5 (0.2%)	no						
* SFTPB-MPZL2*	7 (0.3%)	no						
* SFTPB-STAT6*	5 (0.2%)	no						
* TFG-GPR128*	7 (0.3%)	no						
**Lung squamous cell carcinoma (*N*** = **2426)**								
**Fusions**	***N*** **(%)**	**Kinases**						
* AC011997.1-LRRC69*	25 (1.0%)	no						
* ACTG1-ANXA1*	6 (0.2%)	no						
* ALDOA-ANXA1*	7 (0.3%)	no						
* ARL15-NDUFS4*	5 (0.2%)	no						
* CRHR1-KIAA1267*	39 (1.6%)	no						
* EEF2-ANXA1*	5 (0.2%)	no						
* FGFR3-TACC3*	6 (0.2%)	FGFR3	erdafitinib	Small molecule	pan-FGFR inhibitor	Bladder cancer	1	3B
			fexagratinib	Small molecule	pan-FGFR inhibitor	All solid tumours	4	4
* KIF26B-SMYD3*	6 (0.2%)	no						
* KRT5-ANXA1*	9 (0.4%)	no						
* KRT6A-ANXA1*	5 (0.2%)	no						
* MYH9-COL1A1*	8 (0.3%)	no						
* NDUFA4-PHF14*	5 (0.2%)	no						
* RPL8-ANXA1*	6 (0.2%)	no						
* RPS6KB1-VMP1*	8 (0.3%)	RPS6KB1						
* S100A9-ANXA1*	5 (0.2%)	no						
* TFG-GPR128*	18 (0.7%)	no						
* TP63-TPRG1*	5 (0.2%)	no						
* TTC6-MIPOL1*	5 (0.2%)	no						
* WASF2-AHDC1*	6 (0.2%)	no						

Fusions data are taken from Campbell JD et al, Nature Genetics 2016 [28] [22]; targeted drug response predictions are taken from OncoKB^TM^ [26, 27] (last accessed on May 31st 2025). The level of evidence of the altered gene as biomarker predicting responsiveness to a drug is reported for the neoplasms with highest evidence and for NSCLC.

*NSCLC* non-small cell lung cancer, *TKI* tyrosine kinase inhibitor.

Levels of evidence (OncoKB):

1. FDA-recognised biomarker predictive of response to an FDA-approved drug in this indication.

2. Standard care biomarker recommended by the NCCN or other professional guidelines predictive of response to an FDA-approved drug in this indication.

3A. compelling clinical evidence supports the biomarker as being predictive of response to a drug in this indication.

3B. standard care or investigational biomarker predictive of response to an FDA-approved or investigational drug in another indication.

4. Compelling biological evidence supports the biomarker as being predictive of response to a drug. Most, but not all, associations in a specified cancer type that are OncoKB Levels 1, 2 or 3A will propagate as Level 3B in other cancer types; level 4 alterations do not propagate to other indications (https://www.oncokb.org/faq).

Large-scale copy number alterations (CNAs) may affect several chromosomal arms [22] and some cases of chromothripsis are reported [22, 27]. Amplifications most often involve *NKX2-1* (encoding the Thyroid Transcription Factor 1—TTF1), *MYC*, *TERT*, *MCL1*, while deletions mainly affect *CDKN2A*, *B2M*, *SMAD4* [22, 27, 28] (Table 3).

**Table 3:** Cancer genes focal amplifications and deletions in lung adenocarcinoma, lung squamous cell carcinoma and lung adenocarcinoma without actionable mutations and potentially active targeted drugs.

Cancer gene	OncoKB^TM^ Gene type	Function or pathway	Amp LUAD	Amp LUSC	Del LUAD	Del LUSC	Drug	Drug class	Mechanism of action	OncoKB^TM^ leveled tumour type	OncoKB^TM^ highest level of evidence	OncoKB^TM^ level of evidence in LUAD
***AKT1***	Onc	PI3K/AKT/mTOR		yes			none					
***ARID2***	TSG	SWI/SNF chromatin-remodelling complex			yes		none					
***B2M***	TSG	beta2-microglobulin			yes	yes	none					
***BCL2L1***	Onc, TSG	apoptosis		yes			none					
***CCND1***	Onc	cell cycle	yes	yes			none					
***CCND3***	Onc	cell cycle	yes				none					
***CCNE1***	Onc	cell cycle	yes	yes			lunresertib + camonsertib	SM	PKMYT1 inhibitor + ATR inhibitor	Ovarian and endometrial ca.	3A	4
										All solid tumours	4	
							BLU-222		CDK2 inhibitor	All solid tumours	4	4
							lunresertib		PKMYT1 inhibitor	All solid tumours	4	4
***CDK4***	Onc	cell cycle	yes				abemaciclib, palbociclib	SM	CDK4/6 inhibitor	Liposarcoma	4	/
***CDK6***	Onc	cell cycle		yes			none					
***CDKN2A***	TSG	cell cycle			yes	yes	none					
***CREBBP***	TSG	transcriptional co-activator HAT				yes	none					
***EGFR***	Onc	RTK	yes	yes			cetuximab, panitumumab (±chemo)	MoAb	anti-EGFR MoAb	Esophagogastric cancer	3A	3B
							lapatinib	SM	EGFR- and ERBB2-inhibitor	Glioma	4	/
***ERBB2***^a^	Onc	RTK	yes	yes			trastuzumab deruxtecan	ADC	HER2-targeted ADC	All solid tumours	3A	3A
							trastuzumab, pertuzumab, margetuximab, trastuzumab emtansine, lapatinib, neratinib, tucatinib (±other drugs)	various	Anti-HER2	Breast cancer	1	3B
***FAT1***	TSG	cadherin superfamily member				yes	none					
***FGFR1***	Onc	RTK	yes	yes			none					
***FOXA1***	Onc, TSG	pioneer factor recruiting AR and ER		yes			none					
***FOXP1***	Onc, TSG	transcription factor				yes	none					
***IGF1R***	Onc	insulin-like growth factor receptor		yes			none					
***KAT6A***	Onc	HAT	yes				none					
***KDM5A***	Onc	HMT		yes			none					
***KDM6A***	TSG	histone demethylase				yes	none					
***KMT2C (MLL3)***	TSG	HMT				yes	none					
***KRAS***	Onc	RAS/RAF/MEK/ERK	yes				none					
***MAPK1***	Onc	RAS/RAF/MEK/ERK	yes	yes			none					
***MCL1***	Onc	Apoptosis	yes	yes			none					
***MDM2***	Onc	p53	yes	yes			brigimadlin	SM	MDM2-p53 antagonist	Biliary tract ca., liposarcoma	3A	4
										All solid tumours	4	
							milademetan	SM	inhibitor of p53-MDM2 binding	Intimal sarcoma	3A	3B
										Liposarcoma	4	
***MECOM***	Onc	TF in hematopoietic stem cells	yes				none					
***MET***	Onc	RTK	yes				capmatinib, tepotinib	SM	MET inhibitor	NSCLC	2	2
							crizotinib	SM	multi-kinase inhibitor	NSCLC	2	2
							telisotuzumab vedotin	ADC	anti-MET ADC	NSCLC	3A	3A
***MYC***	Onc	TF regulating many cellular processes	yes	yes			none					
***MYCL1***	Onc	TF of the MYC oncoprotein family	yes	yes			none					
***NF1***	TSG	RAS/RAF/MEK/ERK				yes	none					
***NFE2L2***	Onc	KEAP1/CUL3/NFE2L2 (oxidative stress response)		yes			none					
***NKX2-1***	Onc	TF of lung and thyroid lineages	yes				none					
***NSD3 (WHSC1L1)***	?	HMT	yes	yes			none					
***PDGFRA-KIT-KDR***	Onc	RTK	yes	yes			none					
***PTEN***	TSG	PI3K/AKT/mTOR				yes	none					
***PTP4A1***	?	protein tyrosine phosphatase		yes			none					
***RB1***	TSG	cell cycle			yes	yes	none					
***REL***	Onc	Member of NF-kB family of TFs		yes			none					
***ROBO1***	TSG	cellular migration and axon guidance				yes	none					
***SMAD4***	TSG	TGF-ß			yes		none					
***SMARCA4***	TSG	chromatin remodelling			yes		none					
***SOX2***	Onc	TF involved in cell fate		yes			none					
***TERT***	Onc	catalytic subunit of telomerase	yes	yes			none					
***YES1***	Onc	TK involved in many cellular functions		yes			none					
***ZNF217***	Onc	TF, transcriptional repressor	yes				none					

Copy number alteration data are taken from Campbell et al. [28]; targeted drug response predictions are taken from OncoKB^TM^ [32, 33] (last accessed on May 31st 2025). The level of evidence of the altered gene as biomarker predicting responsiveness to a drug is reported for the neoplasms with highest evidence and for NSCLC.

*ADC* antibody-drug conjugate, *Amp* amplification, *AR* androgen receptor, *ATR* Ataxia Telangiectasia And Rad3-Related, *CDK* cyclin-dependent kinase, *Del* deletion, *ER* oestrogen receptor, *HAT* histone acetyltransferase, *HER*2 (*ERBB*2) Human Epidermal Growth Factor Receptor 2, *HMT* histone methyltransferase, *MDM2* Mouse double minute 2 homologue, *MET* MET proto-oncogene, receptor tyrosine kinase; MoAb monoclonal antibody, *NSCLC* non-small cell lung cancer, *Onc* oncogene, *PKMYT*1 Protein Kinase, Membrane Associated Tyrosine/Threonine 1, *RTK* receptor tyrosine kinase, *SM* small molecule, *SWI*/*SNF* SWItch/Sucrose Non-Fermentable, *TF* transcription factor, *TK* tyrosine kinase, *TSG* tumour suppressor gene.

^a^Associations with some drugs and tumour types have been omitted because they were not relevant for NSCLC.

Levels of evidence (OncoKB):

1. FDA-recognised biomarker predictive of response to an FDA-approved drug in this indication.

2. Standard care biomarker recommended by the NCCN or other professional guidelines predictive of response to an FDA-approved drug in this indication.

3A. Compelling clinical evidence supports the biomarker as being predictive of response to a drug in this indication.

3B. Standard care or investigational biomarker predictive of response to an FDA-approved or investigational drug in another indication.

4. Compelling biological evidence supports the biomarker as being predictive of response to a drug. Most, but not all, associations in a specified cancer type that are OncoKB Levels 1, 2 or 3A will propagate as Level 3B in other cancer types; level 4 alterations do not propagate to other indications (https://www.oncokb.org/faq).

A negative correlation usually occurs between alterations leading to activation of the same pathway, such as those affecting different RTKs, or RTKs and *KRAS/STK11*, or *ATM* and *TP53* [23, 27, 28], or *MYC* and *MGA* [27]. When mutations of *KRAS* coexist with those in *EGFR*, they tend to confer resistance to EGFR inhibitors [34]. Co-occurrences have also been described, i.e., between *MET* amplifications and *NF1* mutations, or *STK11* and *KRAS* mutations [28], or *MDM2* and *CDK4* amplifications. The latter has been suggested to potentially benefit from combined treatment with *MDM2* and *CDK4* inhibitors [35].

**Fig. 1:**
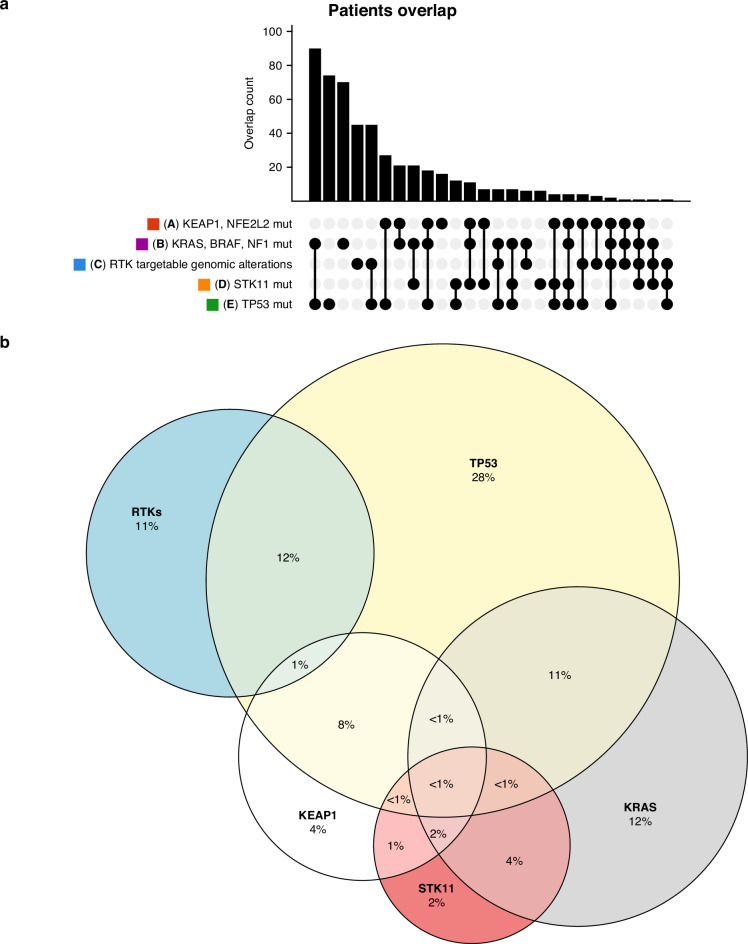
Main genomic alterations in primary lung adenocarcinoma. **a** Counts and co-occurrences of the main genomic alterations in primary lung adenocarcinoma. Downloaded from cBioPortal [189, 190], created from the TCGA PanCancer Atlas case series [27]. Some mutations are grouped by pathway: *KEAP1* and *NFE2L2*; *KRAS*, *BRAF* and *NF1*; RTKs including mutations or fusions of *EGFR*, *ERBB2*, *MET*, *ALK*, *NTRK1-3*, *RET* and *ROS1*. **b** Euler diagram representing the percentages of occurrences and co-occurrences of the main genomic alterations in primary lung adenocarcinoma. The diagram (created with R [191], package 'ggvenn') shows the percentages of genomic alterations affecting the most commonly altered genes in lung adenocarcinoma, either with or without concomitant alterations in other frequently altered genes. RTKs include mutations or fusions of *EGFR*, *ERBB2*, *MET*, *ALK*, *NTRK1-3*, *RET* and *ROS1*. Data are taken from the TCGA PanCancer Atlas case series of lung adenocarcinoma; out of 566 patients in that case series, 83 patients whose tumours do not have any of the considered gene alterations are excluded from the figure. The percentages reported are rough estimates, because the Euler diagram cannot always include all overlapping alterations when considering multiple subgroups.

Figure 1a, b represent the distribution of the main genomic alterations in LUAD, with the main co-mutations.

The pathways most affected by genomic alterations in LUAD include: the RTK/RAS/RAF pathway (76% of cases), the PI3K-mTOR pathway (25%), p53 pathway (63%), cell cycle pathways (64%), the oxidative stress pathway (22%) and chromatin remodelling and RNA splicing pathways (49%) [27].

Smoking history plays a crucial role in shaping the mutational landscape of LUAD. Smokers tend to have higher TMB and an increased rate of cytosine-to-adenine nucleotide transversions [24, 25, 27]. Smoking-related tumours represent around 70% of lung cancers and appear to decline in parallel with the smoking habit reduction in Western world. However, around 30% of LUAD occur in never smokers and are considered a different disease with a definite natural history and a specific treatment. LUAD in never smokers are more common in East Asia and represent the fifth cause of cancer related deaths worldwide, being more common in young females without smoking exposure. It is considered a different and emerging disease, characterised by oncogene addiction because of somatic mutations, chromosomal rearrangements, increased gene copy number, or gene deletions [36]. Mutations in tumour suppressor genes, including *TP53*, *STK11*, *KEAP1*, *NF1*, *SMARCA4*, as well as mutations in *KRAS*, are enriched in smokers. Alterations in RTKs and *PIK3CA* are enriched in non-smokers [24, 27]. The frequency of *EGFR* mutations is higher, and *KRAS* and *BRAF* mutations are lower, in Asian populations compared to Caucasians [29].

About 62% of the 'The Cancer Genome Atlas' (TCGA) LUAD samples had genomic alterations in known driver oncogenes of the RTK/RAS/RAF pathway, including *EGFR*, *ERBB2*, *MET*, *ALK*, *ROS1*, *RET*, *KRAS*, *NRAS*, *HRAS*, *BRAF* and *MAP2K1 (MEK1)*. These have been collectively called 'oncogene-positive' tumours. Another 14% showed amplifications of *ERBB2* or *MET*, or mutations in *RIT1* or *NF1*, eventually leading to activation of the same pathway [27]. Overall, 70–80% of LUAD exhibit alterations in the RTK/RAS/RAF pathway. Common and rare alterations affecting this pathway, along with their therapeutic implications, have been described in excellent reviews [8, 37]. Tumours without such alterations, termed 'oncogene-negative', often have loss-of-function mutations in tumour suppressor genes like *TP53*, *KEAP1* and *NF1* [27].

### Lung adenocarcinoma without actionable genomic alterations within the RTK/RAS/RAF pathway

In an expanded LUAD cohort of TCGA, 15 genes were found to be significantly altered by WES in 'oncogene-negative' tumours [28]. These include regulators of RAS and Rho kinase functions, such as *SOS1*, *RASA1*, *VAV1* and *ARHGAP35*, along with amplifications involving *FGFR1/WHSC1L1*, *PDGFRA/KIT/KDR* and *MAPK1 (ERK2)*. Overall, 20–30% of LUADs lacked RTK/RAS/RAF pathway alterations (RPA) at WES and were termed RPA(-)_E_ cases [38]. WGS identified RPA in 33% of these tumours, which were missed by WES due to technical challenges or low tumour purity [38]. These included KRAS mutations, amplifications of *ARAF*, *EGFR*, *MAPK1* and *SOS1*, deletions of *RASA1* and *NF1* and *NRG1* fusions/mutations.

Among the remaining RPA-negative cases at WGS, or RPA(-)_G_, mutations in tumour suppressor genes such as *TP53*, *STK11*, *KEAP1* and *SMARCA4* were identified, along with mutations in *ESR1*, *BLM* and *FOXO3*, deletions in *SETD2* and amplifications involving *NKX2-1*, *KAT6A*, *CCNE1*, *MDM2*, *MYC*, *MCL1* and *MYCL*. In noncoding regions, including promoters, enhancers and transcription factor-binding sites, mutations were found near *ILF2*, *CUL2* and *TSN*. Mutations in the promoter of *ILF2*, leading to its overexpression, can affect DNA repair and resistance to DNA-damaging agents [39]. RPA(-)_G_ tumours exhibited a high TMB. They also displayed complex structural variants [38], leading to gene amplification and overexpression.

Overall, deep genomic characterisation of RPA(-)_G_ LUADs shows that they are heterogeneous but share some common biological features, including enrichment for *TP53*, *KEAP1* and *SMARCA4* mutations and a high TMB. It remains unclear whether these tumours represent a distinct biological entity or rely on rarer mechanisms (e.g., epigenetic) of activation of the RTK/RAS/RAF pathway. Candidate drivers include *ILF2* mutations, amplifications of genes acting downstream of *RAS/RAF,* such as *MYC*, or loss of tumour suppressors [38].

Rare fusions involving genes, such as *EGFR*, *FGFR*, *MET*, *HER2*, *BRAF*, *NRG* and others were also observed in a small subset of treatment-naïve NSCLC cases, potentially offering new therapeutic options [40].

**Fig. 2:**
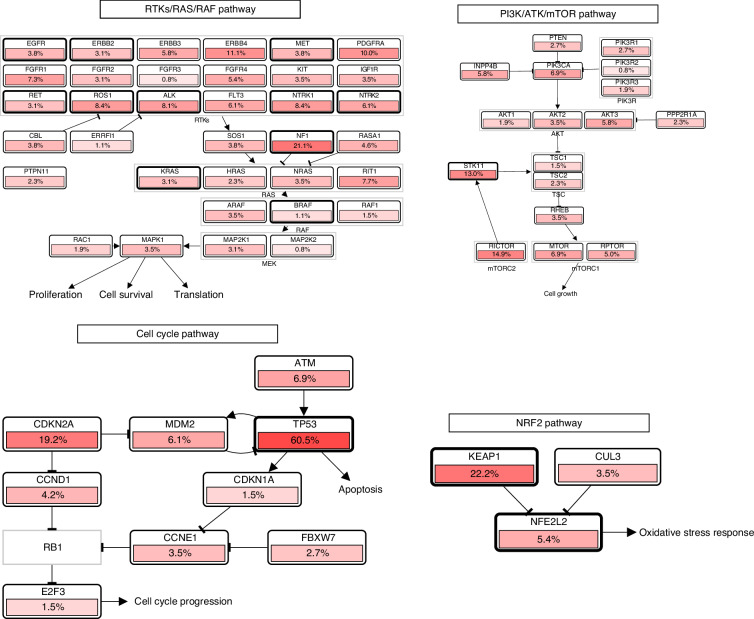
Gene alterations in the pathways more frequently altered in primary lung adenocarcinoma lacking targetable driver alterations in the RTK/RAS/RAF pathway. Figure downloaded from cBioPortal, representing data from the TCGA PanCancer Atlas case series, excluding cases with mutations in *EGFR*, *ERBB2*, *MET*, *KRAS*, *BRAF*, or fusions of *ALK*, *NTRK1-3*, *RET*, *ROS1* (any alterations reported for these genes are represented exclusively by CNAs).

Figure 2 represents the gene alterations in the pathways more frequently altered among LUADs in the TCGA case series [27], considering only cases without actionable driver genomic alterations in the RTK/RAS/RAF pathway.

### Genomic profiling of lung squamous cell carcinoma

The genomic profile of LUSC differs significantly from that of LUAD [28]. LUSC has a high rate of genomic alterations, including mutations, rearrangements and CNAs. According to a TCGA study, frequently mutated genes include *TP53* (mutated in about 90% of cases) and *CDKN2A* (about 70% of cases), as well as *PTEN*, *PIK3CA*, *KEAP1* and *RB1* [26] (a more comprehensive list is provided in Table 1). Further significantly mutated genes include, among others, *HRAS*, *EGFR*, *SMARCA4* and *BRAF*, though the spectrum of EGFR mutations in LUSC differs from that in LUAD.

Amplifications are commonly found in genes like *SOX2*, *FGFR1*, *CCND1*, *PDGFRA*, *EGFR* and *MYC*, while deletions affect *CDKN2A*, *FOXP1* and *PTEN* (a more comprehensive list is provided in Table 3). Although LUSC shows a high number of somatic rearrangements, actionable gene fusions are rare, with only one *NTRK2-TP63* fusion reported in the TCGA series [28].

## NSCLC transcriptomic profiling

Gene expression profiling can distinguish the histological types of lung cancer [41], identify subclasses [41–44] and stratify patients according to prognosis [43–45]. Integrating gene expression profiling with the mutational status of key genes allows further refinement of LUAD’s subclassification [46].

The TCGA study identified three transcriptional subtypes of LUAD: the terminal respiratory unit (TRU), proximal-inflammatory (PI) and proximal-proliferative (PP) [27]. The TRU subtype is enriched with *EGFR* mutations and RTKs fusions, while PI is characterised by *NF1* and *TP53* co-mutations, and PP is associated with *KRAS* mutations and *STK11* loss. The PI and PP subtypes are typically found in smokers and show higher TMB, while TRU is more common in non-smokers and women.

In an enlarged TCGA case series, five transcriptional subtypes (S1–S5) of LUAD were identified via consensus clustering and their features were investigated by integrating multiple data sources [47]. S5 is closely related to the TRU subtype and S4 to the PP subtype, while the PI subtype was split into S1, S2 and S3. Pathway analysis revealed distinct characteristics: S1 had a low immune/inflammatory signature, S2 was enriched in epithelial-mesenchymal transition (EMT) and cell-adhesion signatures, S3 had high immune/inflammatory and proliferation signatures, S4 showed high proliferation, and S5 had a low proliferation signature linked to longer survival. Each subtype was associated with specific gene alterations: *EGFR* in S2 and S5, *KRAS*, *STK11* and *KEAP1* in S4, *TP53* and *NF1* in S3. Vulnerability data highlighted alterations of *CDK4* in S3 and of *CDK6* and *CCND3* in S4, making them potentially sensitive to CDK4/6 inhibitors. In addition to the immune/inflammatory signature and CD274 (PD-L1) amplification and overexpression, S3 exhibited frequent *MET* amplification and overexpression, suggesting a combination treatment of MET inhibitors and ICIs, as well as a combination of CDK4/6 inhibitors and ICIs. The S3 subtype was found to be a stronger predictor of progression-free survival than PD-L1 expression in an independent cohort of LUAD patients treated with ICIs, highlighting its potential clinical utility as a predictive marker. In contrast, mutations in KEAP1 and/or STK11, enriched in the S4 subtype, have been associated in several studies with resistance to anti-PD-1 and anti-PD-L1 [48–52], which can be overcome by using combinations of these drugs with an anti-CTLA4 [53]. Indeed, alterations in KEAP1 and/or STK11, which are especially frequent in KRAS-mutated tumours, promote an immunosuppressive tumour microenvironment, enriched in suppressive myeloid cells and depleted in CD8+ cytotoxic T cells, but with relative sparing of CD4+ effector T cells. The latter are sensitive to anti-CTLA4, and the combination of an anti-CTLA4 with an anti-PD-1 or anti-PD-L1 induces suppressive myeloid cells reprogramming into inducible nitric oxide synthase-expressing tumoricidal phenotypes and recruits circulating neutrophils [53]. Co-mutations in KRAS or TP53 can further affect these phenotypes [54, 55].

For LUSC, four subtypes were identified: primitive, classical, secretory and basal, each with distinct genetic profiles, prognosis and normal cell type correspondence [26, 56]. They are characterised by enrichment of genes involved in proliferation, metabolism, immune response and cell adhesion processes, respectively.

The classical subtype is marked by overexpression of SOX2, PIK3CA and the ΔN-isoform of p63 and by alterations in *KEAP1*, *NFE2L2* (encoding NRF2) and *PTEN*. The primitive subtype shows frequent *RB1* and *PTEN* alterations, and the basal subtype *NF1* alterations.

Transcriptomic profiling holds promise for predicting treatment responses and outcomes. Numerous transcriptomic predictors have been developed to predict responses to ICIs [57], with some specifically applied to NSCLC [58, 59]. A notable example is the T-effector/interferon-γ-associated gene expression signature, which reveals immune activation through the levels of transcripts of PD-L1, CXCL9 and IFNγ. High expression of this signature correlates with benefit from atezolizumab in first- and second-line therapy of NSCLC, particularly in patients lacking RTK/RAS/RAF genomic alterations [60–62].

Other gene signatures focus on identifying tumours with significant activation of specific pathways. For instance, a 46-gene signature predicts the activation of the *KEAP1*/*NFE2L2*(NRF2) antioxidant pathway [63]. The associated K1N2 score not only reliably indicates the presence of *KEAP1/NFE2L2* mutations but also outperforms mutational analysis in predicting patient survival.

## Proteogenomics of NSCLC and pathways activation and targeting

Proteogenomic studies integrate genomic and proteomic data, alongside other omics like DNA methylation, RNA-seq and post-translational modifications (PTMs) such as protein phosphorylation, to provide a functional analysis of the cellular signalling networks and identify relevant therapeutic targets [21]. Most proteogenomic studies in NSCLC [64] are not specific for tumours with or without actionable genomic alterations but identify subgroups of tumours that are related to these categories.

### Proteogenomic analysis of lung adenocarcinoma

Proteomics provides quite different information from transcriptomics on cellular phenotypes. The strength of the correlation between mRNA and protein levels for a given gene depends, at least in part, on post-transcriptional (e.g., splicing, microRNA-mediated regulation) and post-translational (e.g., ubiquitination, protein degradation) mechanisms. These regulatory processes can vary across tumour subgroups, influencing signalling networks and tumour behaviour. In LUAD, gene-specific mRNA–protein correlation patterns have been used to identify dysregulated genes in tumours with early recurrence compared to those that remain relapse-free [65].

Phosphoproteomic analyses of LUAD have revealed that activation of key growth pathways like RAS/MAPK and PI3K/AKT/mTOR are only partially driven by genetic alterations in the same pathways [27]. Drugs targeting specific proteins within a pathway may also be effective when the pathway is activated by alterations in other proteins, even if the direct target is not mutated. One such example is the CDK4/6 inhibitor palbociclib, which has shown efficacy in NSCLC patients with *KRAS* mutations [66] but performed poorly in cases with *CDKN2A* mutations or in LUSCs with *CCND1-3*/*CDK4* amplifications [67]. Such drug sensitivities can be attributed to signalling network rewiring resulting from the genetic and epigenetic alterations of a specific tumour [68].

A comprehensive proteogenomic study of 110 treatment-naïve LUAD samples from patients of different ancestries identified four distinct multi-omics clusters (C1–C4), which partly overlap with the previously identified transcriptomic subtypes PI, PP and TRU, but subdivide the second into two distinct clusters [69]. These clusters are characterised by different genetic and molecular features. C1 includes cases enriched with *TP53* mutations, CpG island methylator phenotype (CIMP)-high status and a high TMB, C2 is characterised by wild-type *TP53* and *EGFR* and intermediate CIMP, C3 is enriched for Vietnamese patients and *STK11* mutations, while C4 is enriched with *EGFR* mutations and *EML4-ALK* fusions, primarily affecting Chinese and female patients. Each cluster also displays distinct pathway activations, such as immune signalling in C1, Rho GTPases and haemostasis/platelet in C2, histone deacetylase and cell cycle in C3 and MAPK1/MAPK3, MECP2 regulation, surfactant metabolism and chromatin organisation in C4. The study identified new potentially oncogenic gene fusions, some of which activate oncogenes like *PTK2*, *PDGFRA* and *GSK3B*, or disrupt tumour suppressors like *STK11*, *STK4* and *ATM*. Phosphoproteomic analyses linked these fusions to specific activated pathways, providing insights into potential therapeutic targets. Analyses of paired tumours and normal tissues identified increased expression and altered PTMs of proteins linked to cell cycle progression, glycolysis and MYC targets in LUAD samples. NPM1 and MKI67 showed increased phosphorylation and Histone 2B and EP300 increased acetylation in tumours and five proteins (GFPT1, BZW2, PDIA4, P4HB and PMM2) were consistently upregulated, suggesting their potential as biomarkers or drug targets.

CIMP-high tumours, frequent in the C1 cluster, are characterised by CpG islands hypermethylation in the promoter regions of multiple genes, leading to reduced expression of several genes associated with lung cancer development and prognosis, such as *CLDN18*, *ANK1* and *PTPRCAP*. Potential therapeutic approaches for CIMP-high tumours include DNA demethylating agents and histone deacetylase inhibitors, though their efficacy in lung cancer remains unproven.

Proteogenomic analyses from the same study revealed several therapeutic vulnerabilities in LUAD, including the upregulation of mismatch repair and DNA damage response proteins in *TP53*-mutated tumours. These may predict sensitivity to ICIs and to PARP inhibitors, respectively, though results of PARP inhibitors in NSCLC have been unsatisfactory [70–76]. Additionally, *TP53* mutations were associated with increased expression of EZH2, a lysine methyltransferase that methylates histone H3, inducing chromatin condensation and resistance to DNA-damaging agents, suggesting a potential role for EZH2 inhibitors [77]. Other driver mutations, such as in *SMARCA4* and *STK11*, were linked to increased expression or phosphorylation of SMAD2 and SMAD4, proteins involved in the TGF-β pathway, suggesting potential sensitivity to the TGF-βR1 inhibitor vactosertib [78]. *KEAP1* mutations lead to increased levels of NRF2, which is involved in antioxidant responses through activation of mTOR signalling. Inhibitors targeting TORC1/2 have shown preclinical activity in lung cancer models with KEAP1/NRF2 pathway alterations [79]. *KRAS* mutations were linked to the activation of SOS1 and early data suggest that SOS1 inhibitors may enhance the efficacy of KRASG12C inhibitors in LUAD [80]. The SHP2 protein tyrosine phosphatase, encoded by PTPN11, is frequently phosphorylated in EGFR mutant and in ALK fusion-positive LUAD, leading to activation of the MAPK pathway. SHP2 inhibitors have shown promise in preclinical studies of *EGFR*-mutant and *ALK* fusion-positive tumours with activation of MAPK pathway [81, 82]. The study identified several hyperphosphorylated kinases that are known drug targets in other cancers. These included PRKCD in *KRAS*-mutant tumours, BRAF in *TP53*-mutant tumours and WEE1 in *EML4-ALK* fusion-positive tumours. Additionally, 27 other putatively druggable hyperphosphorylated kinases, whose inhibitors have not yet been approved by the FDA, were also identified [69].

In the same study, the immune landscape of LUAD was classified into three clusters: hot-tumour-enriched (HTE), cold-tumour-enriched (CTE) and normal adjacent tissue (NAT)-enriched. HTE tumours show upregulation of members of multiple immune-related pathways, including PD-1, PD-L1, CTLA4, FOXP3, and IDO1, suggesting the utility of combination immunotherapy. In contrast, CTE tumours showed upregulation of epithelial barrier components, contributing to immune suppression, while NAT-enriched tumours showed intermediate immune signatures. *STK11* mutant tumours show strong immune downregulation associated with a neutrophil degranulation signature. The study identified several cancer-testis antigens that are immunogenic and could be useful to develop tumour vaccines [69].

A proteogenomic study on 103 LUAD cases in Chinese patients revealed that 50% had *EGFR* mutations. The study identified three proteomic subtypes: one linked to cellular environment and metabolism pathways, a mixed subtype with most *EGFR* mutations and a subtype enriched in proliferation with a higher TMB. In total, 11 potential drug targets were identified, including IMPDH2 and GAPDH, which are FDA-approved [83]. Despite East-Asian LUAD often occurs in non-smokers with *EGFR* mutations, *TP53* mutations were found in a third of cases in Taiwan patients and were associated with activation of proteins involved in DNA repair, presenting further therapeutic opportunities [84]. The APOBEC mutational signature, linked to immunotherapy benefits, was common in younger women without *EGFR* mutations and was associated with the activation of CDK1, CDK2 and Aurora Kinase B as potential targets. Additionally, matrix metalloproteinases, especially MMP11, were linked to poor survival, representing another therapeutic target [84].

In a proteogenomic study on 87 LUAD cases in the U.S., multi-omics clustering confirmed the transcriptomic subtypes TRU, PI and PP [85]. While the TRU subtype is enriched in *EGFR* mutations, the PI subtype showed enhanced IFN-γ signalling and PD-L1 and CTLA4 expression, making it potentially responsive to ICIs. The PP subtype is characterised by activation of several CDKs and of MAP2K7 and by metabolic alterations affecting glycolysis and glutaminolysis. As SMARCA4 inactivation is known to be synthetic lethal with CDK4, PP tumours with *SMARCA4* mutations and high CDK4 activity may respond well to CDK4/6 inhibitors. They are also potentially vulnerable to glutaminase inhibitors in *STK11-KEAP1-KRAS*-mutant cases or to stearoyl-coenzyme A desaturase inhibitors in combination with ferroptosis inducers in *STK11-KEAP1* co-mutant cases.

Proteogenomic studies can provide valuable insights into the molecular characteristics and therapeutic opportunities in lung cancers related to specific aetiological factors. One such study focused on 169 never-smoking females from the Xuanwei area in China, where exposure to coal smoke is the primary cause of LUAD [86]. The study identified benzo[a]pyrene, a polycyclic aromatic hydrocarbon, as the main carcinogenic agent. LUADs linked to this carcinogen exhibited unique molecular features, including specific EGFR G719C/A/D/S (G719X) mutations, which were present in 20% of the cohort. These mutations were associated with upregulation of components of the MAPK signalling pathway, such as MAP2K2 (MEK) and MAPK3 (ERK1), as well as kinases involved in cell cycle regulation, including CDK2, AURKB, CSNK1A1 and CDK4. Notably, these kinases are targets of drugs that are either already approved or currently in clinical development. Afatinib and osimertinib are recommended as the preferred first-line therapies for LUADs with these specific EGFR mutations [11]. However, the study suggests that combining these agents with inhibitors targeting the upregulated downstream kinases may provide a strategy to overcome resistance and improve treatment outcomes.

Another area of proteogenomics investigation concerns pre-invasive or minimally invasive lesions, to shed light on mechanisms of carcinogenesis, develop diagnostic and prognostic tools and identify therapeutic targets [87–89].

### Lung adenocarcinoma without actionable genomic alterations

Some proteogenomic studies have been conducted specifically on LUADs lacking clinically actionable genomic alterations. One such multi-omics study focused on non-coding regions of DNA [90]. A significant finding was the lack of enhancer activity in the *MAML2* gene, linked to translocations (forming fusion genes such as *CRTC1-MAML2*, a known oncogenic driver in mucoepidermoid carcinoma), enhancer mutations, DNA methylation or histone acetylation changes, which downregulated MAML2. This led to downregulation of members of the NOTCH and WNT/β-catenin pathways, while ERBB2 was overexpressed. Patients with low CD302, FAT4 and FOXN3 expression, associated with MAML2 downregulation, had worse overall survival and showed upregulation of molecules like PLK1, UBE2C and LYPD3, which might represent therapeutic targets. Epigenetic therapies could potentially restore MAML2 expression in some cases.

A study on Korean patients with EGFR- and ALK-wildtype LUAD revealed elevated oestrogen receptor (ER) signalling, especially in never-smoker with *STK11* mutations, while *KRAS* mutations were associated with elevated ER signalling regardless of smoking status [91]. Deletions in chromosomes 14 and 21, DNA hypomethylation of genes *LLGL2* and *ST14* and *SRC* overexpression were also associated with increased ER signalling. The SRC inhibitor saracatinib showed activity in *STK11*-mutant and especially in *STK11/ERBB2* co-mutated LUAD cell lines, representing a potential therapy in this setting, while selective ER modulators like tamoxifen were inactive.

A proteogenomic study of 99 never-smoking Korean patients with EGFR- and ALK-wildtype LUAD identified four molecular subgroups with distinct clinical outcomes, based on previously defined tumour and microenvironment signatures applied to transcriptome and proteome data [92]. The Proliferation-high (P) subgroup, with a worse prognosis, is marked by *TP53* and *ARID1A* mutations, upregulation of proliferation-related genes, high Ki67 levels and low immune activation. It is enriched for multiple actionable kinases, including CDK2, CDK5, polo-like kinases and ATR and shows greater dependency on *CDK9* at vulnerability screens. The Immune-high (I) subgroup shows high immune cell infiltration, particularly B-cells, and upregulation of immune checkpoints (e.g., PD-1, TIGIT, CTLA4), cytokines (e.g., CXCL13, CD27) and chemokines (e.g., CCL5). It shows dependency on *TRAF2*, a mediator of resistance to ICIs [93]. The Angiogenesis-high (A) subgroup features *TP53* and *KRAS* co-mutations and upregulation of proangiogenic factors (FGF2, CXCL12, PDGFB, LGALS3), often secreted by stromal cells. It showed pronounced dependency on *GRB2*, encoding an adaptor protein for RTKs. Lastly, the Metabolism (M) subgroup shows upregulation of metabolic enzymes involved in oxidative phosphorylation, lipid and carbon metabolism, upregulation of signalling molecules such as ERBB3, ICK and ARAF, partial CD8+ T-cell suppression and high expression of CERS4, an enzyme involved in sphingolipid metabolism and linked to anti-PD-1 responses [94]. It shows dependency on metabolic genes such as *ACACA*, involved in mitochondrial fatty acid synthesis. According to the PRISM repurposing dataset [95], potentially active drugs include digitoxin and the histone deacetylase tacedinaline in subgroup P, the lysophosphatidic acid receptor antagonist KI16425 in subgroup I, the growth hormone secretagogue ibutamoren (MK-677) in subgroup A and the 3-phosphoglycerate-inhibitor veterinary anthelmintic clorsulon for the M subgroup. These findings reveal the molecular diversity within EGFR- and ALK-wildtype LUADs in never smokers and suggest subgroup-specific vulnerabilities for tailored therapies.

### KRAS mutant lung adenocarcinoma

Some proteogenomic studies have focused on *KRAS*-mutant LUADs. A proteomic study considering only *EGFR* wildtype cases found that approximately one-third of *KRAS*-mutant LUADs had higher ERK pathway activation compared to the *KRAS* wildtype counterpart [96]. Cross-talks were observed between KRAS effectors and the AKT/mTOR pathway, along with correlations to RTKs phosphorylation. Additionally, 18% of *KRAS*-mutant tumours exhibited increased phosphorylation of ER alpha, supporting potential treatment strategies targeting multiple pathways.

Further integrative analysis classified *KRAS*-mutant tumours into three subgroups based on co-mutations with *STK11*, *TP53* and *CDKN2A/B*, each showing distinct genomic characteristics and potential treatment vulnerabilities, such as sensitivity to HSP90 inhibitors in *KRAS/STK11* co-mutated LUAD cell lines [54]. ICIs could be effective for tumours in the *KRAS/TP53* co-mutated subgroup, which shows expression of PD-1/PD-L1 and CTLA4.

Finally, an analysis integrated cancer cell line dependencies, gene actionability and patient genomic data to identify EGLN1 as a therapeutic target, especially in KRAS-mutated LUAD [97].

### TTF1-negative lung adenocarcinoma

TTF1-negative LUADs have fewer actionable mutations and poorer outcomes. A proteogenomic study revealed that these LUADs are enriched with *KEAP1* mutations, leading to increased NRF2 expression, which may be a potential therapeutic target [98, 99]. The study also found increased expression of DNA repair enzymes (e.g., CHK1), cell cycle molecules (e.g., cyclin B1) and reduced MAPK and PI3K/mTOR signalling. LUAD cell lines with low TTF1 expression were more sensitive to DNA repair-targeting drugs like PARP and ATM inhibitors but showed reduced sensitivity to paclitaxel and pemetrexed. Additionally, TTF1-negative LUAD cell lines often overexpress SRGN, encoding the proteoglycan serglycin, which promotes PD-L1 expression, proinflammatory cytokine production and increased tumour invasiveness and represents a potential therapeutic target [100].

### Proteogenomic analysis of lung squamous cell carcinoma

A multi-omics study of LUSC identified three primary proteomic subtypes: inflamed, redox and mixed [101]. Each subtype has distinct characteristics and potential therapeutic implications. The inflamed subtype is characterised by an intense inflammatory infiltrate, involving neutrophils, lymphocytes, monocytes, regulatory T cells or myeloid-derived suppressor cells. These tumours show elevated expression of neutrophil-associated proteins, extracellular matrix proteins and PD-1 and tend to harbour fewer mutations in key genes and fewer CNAs compared to other subtypes. They often present tertiary lymphoid structures (TLSs), which are associated with better prognosis. Combination immune therapies could be particularly effective for the inflamed subtype, and it has been suggested that adding an anti-CD33 drug, such as gemtuzumab ozogamicin, might enhance therapeutic responses by eliminating immunosuppressive myeloid cells. The redox subtype is distinguished by alterations in metabolic oxidation-reduction processes. It shows elevated expression of aldo-keto reductase and alcohol dehydrogenase enzymes, along with high rates of CNAs, particularly amplifications in chromosome regions 3q2 (harbouring *TP63*, *SOX2* and *PIK3CA*) and 2q3 (containing *NFE2L2*). Mutations in *NFE2L2* and *KEAP1* are also prevalent, with 84% of redox tumours exhibiting alterations in these genes. Based on public RNA interference and CRISPR knockout screenings data, three genes—*TP63*, *PSAT1* and *TFRC*—were identified as promising therapeutic targets for this subtype. *TP63* encodes the ΔNp63 protein, which has oncogenic functions and regulates glutathione metabolism, while *PSAT1*, whose expression is induced by NRF2, is involved in serine biosynthesis and associated with poor prognosis. *TFRC* encodes the transferrin receptor 1, involved in ferroptosis, a form of programmed cell death controlled by NRF2. This subtype has metabolic vulnerabilities, including altered serine biosynthesis, glycolysis and reactive oxygen species (ROS) production, potentially making these processes promising therapeutic targets. The mixed subtype, the smallest of the three, is characterised by expression of proteins linked to the WNT/β-catenin pathway and an enrichment of *APC* mutations.

In a proteomic study comparing LUSC to LUAD, a shift from cap-dependent to cap-independent translation was highlighted as a key feature of LUSC [102]. Under stress conditions like hypoxia or ROS, mTOR activity decreases, leading to suppression of cap-dependent translation and an increased reliance on cap-independent translation. This process facilitates the production of oncogenic proteins like HIF1α, MYC, VEGFA and BCL-2, which support tumour growth, angiogenesis and cell survival.

In another thorough proteogenomic study, multi-omics clustering identified five subtypes of LUSC: Basal-Inclusive (B-I), Epithelial-to-Mesenchymal Transition-Enriched (EMT-E), Classical, Inflamed-Secretory (I-S) and Proliferative-Primitive (P-P) [103]. The B-I subtype displayed overexpression of TROP2, a target of antibody-drug conjugates like sacituzumab govitecan, while the EMT-E subtype was marked by activation of targetable proteins such as PDGFRB and ROR2. Importantly, the study identified loss of cell cycle inhibitors, such as *CDKN2A*/p16INK4a and *RB1*, due to genetic, epigenetic, or unknown reasons, as a universal feature of LUSC, and amplification of *CCND* or *CDK4/6* genes was also frequent. CDK4/6 inhibitors have shown limited success in LUSC, but a subset of patients with high RB phosphorylation levels might benefit from these drugs, as demonstrated in LUSC cell lines, where RB phosphorylation levels were more predictive of response than alterations in CCND1, CDK2NA and RB1. Several other key signalling pathways and proteins were identified as potential therapeutic targets. These include the NRF2 pathway, frequently activated through mutations in *NFE2L2*, *CUL3*, or *KEAP1* in the classical subtype, but sometimes also in the absence of those mutations and possibly because of CDK5 upregulation. The squamous differentiation marker SOX2, frequently co-amplified with *TP63* in the classical subtype, is generally considered undruggable, but was positively correlated with the chromatin modifiers KDM1A (LSD1), KDM3A and EZH2, whose inhibition leads to SOX2 downregulation. Conversely, LUSC cases with low ΔNp63α expression show upregulation of survivin (BIRC5) and are potentially responsive to survivin inhibitors [103]. Expression of the immune checkpoints PD-1, PD-L1, CTLA4 and IDO1 is prominent in the I-S subtype, predicting responsiveness to ICIs. Additionally, upregulation of Rho GTPase signalling in immune cells and of CSF1R predominantly in macrophages, along with dysregulation of other immune-related proteins, represents further potential targets in the I-S subtype. The analysis of CNAs in this study showed that *WHSC1L1* belongs to the same amplicon as *FGFR1* and may be the critical driver oncogene, thus representing a potential therapeutic target and explaining the failure of anti-FGFR1 drugs in this context. Lastly, LUSC tumours showed upregulation of protein kinases such as EGFR, SRC and MAPK14, and EGFR phosphorylation correlated with ligand abundance rather than with gene amplification [103].

### Proteogenomic studies including multiple histological types of NSCLC

Some proteogenomic studies involved an analysis of both LUAD and LUSC cases. A multi-omics cluster of clusters analysis of 1,023 NSCLC cases from TCGA identified nine tumour subtypes, three with predominantly LUSC and six with predominantly LUAD cases [104]. LUSC subtypes showed increased *SOX2* amplification and expression, as well as p63 and KRT5/6 overexpression and more *PTEN* losses, while LUAD subtypes had higher expression of NKX2-1 and KRT7 and more *STK11* alterations with decreased expression. LUAD subtypes also showed elevated mTOR and MAPK pathways phosphorylation, suggesting potential therapeutic responsiveness. Immune checkpoint activation and cancer-testis antigen expression were present in both LUSC and LUAD subtypes, offering therapeutic opportunities.

In a study of 141 NSCLC samples, including all major histological types, six proteome subtypes were identified [105]. Subtypes 1–4 mostly included LUADs, subtype 5 included neuroendocrine neoplasms and subtype 6 comprised LUSCs. Subtypes were further analyzed based on immune cell infiltration, metabolic pathways and mutations. Subtypes 2 and 3 had higher immune infiltration, subtype 5 had the highest proliferation rates, and subtype 1 had the lowest. The predominant mutations affected *EGFR* in subtype 1, *STK11*, *KEAP1* and *SMARCA4* in subtype 4, *RB1* in subtype 5 and *TP53* in subtype 6. This aligns with the network analysis findings of metabolic pathways activation in subtype 4, E2F1/MYC signalling in subtype 5 and p53 signalling in subtype 6. While subtypes 2 and 3 showed high levels of T cell and B cell infiltrates, respectively, the immune-cold subtypes 4 and 6 were characterised by low immune activity despite neoantigen expression. These subtypes expressed immune-inhibitory ligands, such as FGL1 (binding to LAG-3) and B7-H4 (binding to activated T-cells), which could be treated with anti-LAG-3 and anti-B7-H4 therapies. Subtype 4, associated with *STK11* mutations, showed activation of mTOR signalling and potential vulnerability to mTOR inhibitors combined with LAG-3/FGL1 checkpoint inhibitors.

An integrative multi-omics analysis of 229 Korean patients with NSCLC identified five molecular subtypes, validated through prior multi-omics studies [106]. The cohort included LUADs (61%), LUSCs (27%) and other histological types (12%) across early and advanced stages. Subtype 1, termed 'metabolic', was predominantly composed of LUAD cases in female patients. This subtype was enriched with *EGFR* and *TP53* mutations, *CDKN2A* copy number loss and frequent whole genome doubling (WGD) events. It also exhibited upregulation of proteins involved in metabolic pathways. Subtype 2, referred to as 'alveolar-like', consisted mainly of LUADs with *EGFR* mutations but low frequencies of *TP53* mutations and WGD events. This subtype displayed activation of IL-33 and NOTCH pathways and was associated with the best survival. Subtype 3, labelled 'proliferative', primarily included LUSCs from male smokers. It had the highest frequency of WGD events, frequent *TP53* and *PIK3CA* mutations and amplifications on chromosome 3q involving *SOX2* and other cancer-related genes. This subtype was enriched in cell-cycle related pathways, including E2F/MYC targets, G2M checkpoint and CDKs. Among the upregulated proteins was XPO1, whose inhibitor selinexor demonstrated antitumor activity in WGD-positive LUSC organoids. Subtype 4, termed 'hypoxic', included all histological types and was associated predominantly with metastatic cases and the poorest prognosis. It was characterised by activation of hypoxia, PI3K-AKT and neutrophil degranulation pathways. This subtype showed upregulation of CSNK2A1 and GSK3B, known to activate the PI3K-AKT pathway, as well as SLK (phosphorylated by CSNK2A1 and involved in apoptosis) and LRRFIP1 (promoting EMT), both conferring a poor prognosis. Subtype 5, called 'immunogenic', showed frequent tumour-infiltrating lymphocytes (TILs)-associated patterns, enrichment in *KRAS* mutations and in immune-related pathways such as TNFα signalling via NF-kB. Analysis of the tumour immune microenvironment revealed three immune clusters: HTE, CTE and NAT-enriched. While HTE tumours were generally associated with better prognosis, this advantage was diminished in cases with regulatory T-cell (Treg) enrichment. Subtype 5 tumours, often enriched in HTE patterns, also frequently exhibited Tregs and neutrophils, reducing survival outcomes. Upregulated immunomodulators in HTE tumours included SLAMF7, associated with the presence of *SMARCA4* mutations and a target of the monoclonal antibody elotuzumab. Furthermore, subtype 5 was characterised by cryptic MHC class I-associated peptides [107], noncanonical neoantigens derived from noncoding transcripts, pseudogenes, or untranslated regions of mRNA. These neoantigens were positively correlated with HTE status and demonstrated prognostic significance. From a therapeutic perspective, subtype 5 showed the largest benefit from adjuvant chemotherapy or chemoradiation, unlike the other subtypes. Additionally, ICIs could provide benefit for this subtype.

## Single-cell analyses assessing tumour and microenvironment heterogeneity

NSCLC is characterised by significant intratumor heterogeneity, affecting prognosis and treatment response. The TRACERx study revealed that, on average, each NSCLC harbours 4.2 truncal and 2.8 subclonal driver mutations, with 77% of tumours showing at least one WGD event and 19% showing at least one subclonal WGD event [108, 109]. In LUAD, mutations in RTKs, MYC and NRF2 pathways are early events under truncal selection. Other mutations in key cancer genes like *STK11*, *TP53* and *KRAS* may act as either truncal or subclonal mutations. Mutations in chromatin remodelling and NOTCH pathways or in genes like *PTEN*, *RUNX1* and *SMAD* are often subclonal and involved in later stages of tumour evolution. In LUSC, subclonal selection frequently affects mutations in a different gene set, including *ATM*, *KEAP1*, *NFE2L2* and *PIK3CA*.

Single-cell RNA sequencing (scRNA-seq) has revealed distinct cellular compositions and interactions in NSCLC subtypes, including different spectra of stromal and immune cells [110] and different expression of immune checkpoint molecules (e.g., TIM3 and TIGIT in LUAD, CD96 in LUSC and LILRB1/2 in both tumour types), highlighting different potential therapeutic targets [111]. A combined analysis including scRNA-seq and bulk RNA-seq identified AT2 cells as dominant malignant cells in LUAD and basal cells in LUSC [112], with distinct oncogenic drivers. NKX2-1 emerged as a key regulator of AT2 cells in LUAD, whereas KLF5 and MYC were identified as key transcription factors in basal cells in LUSC. Other genes overexpressed and with potential oncogenic roles are AZGP1 and S100A13 in AT2 cells in LUAD and PPT1 and KPNA2 in basal cells in LUSC, all promoting cell proliferation and representing potential therapeutic targets.

Single-cell transcriptomic and multiomic analyses, often combined with spatial analysis, are promising tools to improve the prediction of response to ICIs, considering both tumour features and the stromal and immune cells landscape [113]. In patients treated with neoadjuvant nivolumab, scRNA-seq showed lower expression of genes involved in cytolytic programs and upregulation of immune checkpoints in TILs specific for mutation-associated neoantigens, compared with TILs not specific for neoantigens [114]. Furthermore, TILs from patients who achieved a major pathological response (MPR) showed higher expression of genes associated with effector and memory functions and lower expression of exhaustion markers compared to TILs from patients who did not achieve an MPR. Beyond highlighting the high tumour and stromal/immune cell heterogeneity in LUAD, spatial analyses found increased T-regulatory cells and decreased cytotoxic T cells and antigen-presenting cells in normal lung parenchyma adjacent to the tumour compared to distant parenchyma [115]. A proteomic signature derived from stromal areas has been found to be a stronger predictor of response to ICIs than a signature from the tumour compartment [116]. The presence of activated TLSs [117], composed mainly of germinal centre B cells, effector memory CD4 T cells and follicular helper T cells, as well as the presence of stem-immunity hubs [118], including mainly stem-like TCF7^+^PD-1^+^CD8^+^ T cells, activated CCR7^+^LAMP3^+^ dendritic cells and CCL19^+^ fibroblasts, have been shown to predict responses to ICIs. Moreover, the proportion of collagen type XI alpha 1 chain-positive (COL11A1^+^) cancer-associated fibroblasts (CAFs) was significantly higher in LUAD from non-responders than in responders to neoadjuvant chemo-immunotherapy and non-responders showed an increase in monocytes/macrophages and dendritic cells in post-treatment surgical samples compared to baseline biopsies [117]. COL11A1^+^ CAFs co-localise with SPP1^+^ macrophages at tumour borders and their interaction promotes the production of collagen by CAFs, that obstacles the contacts between tumour cells and cytotoxic immune cells, leading to immune exclusion.

## Specific features of metastatic NSCLC

Studies on metastatic NSCLC reveal a different mutational landscape compared to primary tumours, with higher rates of *EGFR* alterations, which could be due to referral bias. An analysis of 860 patients with recurrent or metastatic LUAD, mostly pretreated, identified actionable somatic alterations in 87% of cases [119]. Most alterations involved the RTKs/RAS/MAPK pathway, the PI3K/AKT/mTOR pathway, or *BRCA1/2*. Twelve percent of the tumours lacked any known actionable somatic alterations and were classified as the 'unknown mitogenic driver' subset. They were enriched for alterations in *TP53*, *STK11*, *KEAP1*, *KMT2D* and *PDGFRA*, as well as other alterations related to smoking history.

The TRACERx study revealed that metastatic lesions often harbour unique mutations not found in primary tumours, but only 33% of these are driver mutations, affecting genes like *TP53*, *KMT2D*, *STK11*, *SMARCA4*, *FAT1*, *NF1*, *RBM10*, *PIK3CA*, *ARID1A*, *CUX1*, *FBXW7*, *EGFR*, *ARHGAP35* [120]. Metastasis-unique mutations can arise from therapeutic pressure, such as those in PMS1 linked to platinum-based chemotherapy.

A study of over 2500 samples, including primary and metastatic LUAD, found that metastasis-unique actionable oncogenic alterations occurred in only 4% of metastases and were often linked to resistance mechanisms developed in patients treated with RTK inhibitors [121].

## Targeting alterations in tumour suppressor genes

A critical issue in the treatment of NSCLC is targeting alterations in key tumour suppressor genes. TP53, the most frequently mutated gene in NSCLC [26, 27], exemplifies the complexity of tumour molecular mechanisms [122, 123]. Missense mutations often lead to conformational changes in p53, resulting in varying degrees of loss of function, but in some cases also leading to gain-of-function effects, typically of a non-canonical oncogenic nature, or to increased immunogenicity. Truncating mutations are usually associated with a complete loss of function. Reduced expression and function of p53 may stem from overexpression of its inhibitors MDM2 or MDM4, due to gene amplification or post-translational modifications, or from deletion of CDKN2A, which encodes p14^ARF^, an MDM2 inhibitor. Occasionally, aberrant p53 conformation and function are observed even in TP53 wild-type tumours, either in cancer cells or in stromal cells such as the CAFs supporting tumour growth [123]. Conversely, certain structural p53 mutants may, under specific conditions, regain a normal conformation [124]. Thus, genomic characterisation alone may be insufficient to identify all the alterations in the p53 pathway.

A variety of therapeutic strategies aimed at restoring normal p53 functions are under preclinical and clinical development [122, 123]. In TP53 wild-type tumours, inhibitors of the p53–MDM2 interaction, such as idasanutlin, have thus far yielded limited efficacy and notable toxicity, but newer molecules are currently in development [123]. Other inhibitors, such as kevetrin, which blocks the E3 ligase activity of MDM2 and prevents p53 degradation [125] and the stapled peptide sulanemadlin, which mimics the N-terminal domain of p53 and binds to MDM2 to inhibit its activity [126], are also being studied. Proteolysis-targeting chimeras (PROTACs) [122, 127], bifunctional molecules that simultaneously bind a target protein, such as MDM2 and an E3 ubiquitin ligase to induce proteasomal degradation, are effective at sub-stoichiometric concentrations and are progressing through clinical development. In TP53-mutated tumours, drug development efforts are focused on restoring proper p53 conformation and transcriptional activity. In this context, eprenetapopt is in advanced clinical development with some promising results [128, 129] and recently, rezatapopt, a small molecule that selectively reactivates p53 carrying the Y220C mutation, has demonstrated strong preclinical activity and is now undergoing clinical evaluation [130]. Another class of investigational drugs are those promoting translational readthrough of nonsense-mutant TP53, including certain aminoglycoside and macrolide antibiotics and 5-fluorouridine, which enable ribosomes to bypass premature termination codons and produce a full-length functional protein [123]. Numerous other therapeutic strategies focused on p53 are under clinical investigation, including gene therapy, vaccines, synthetic small interfering RNA oligonucleotides, bispecific antibodies targeting mutant p53, targeted T cell receptor–T cell therapies and agents that exploit synthetic lethality in tumours harbouring TP53 mutations [122, 123].

Another frequently mutated tumour suppressor gene in NSCLC is *KEAP1*, which is part of the NRF2 signalling pathway. The transcription factor NRF2 is a master regulator of cellular homoeostasis, controlling redox balance and various aspects of cellular metabolism [99]. KEAP1 acts as an adaptor protein, linking NRF2 to the Cullin3–RBX1 E3 ubiquitin ligase complex, leading to NRF2 ubiquitination and proteasomal degradation. This mechanism maintains low levels of NRF2 under non-stressed conditions. Electrophilic compounds and ROS can modify specific cysteine residues in KEAP1, inducing a conformational change that disrupts its adaptor function. As a result, NRF2 accumulates and activates the transcription of stress-response genes to restore cellular homoeostasis. Prolonged NRF2 activation may also result from KEAP1 sequestration in autophagosomes in response to various stimuli.

The NRF2 pathway, through its antioxidant and detoxifying activities, plays a protective role in normal cells by preventing neoplastic transformation and NRF2 agonists are being studied in cancer prevention [131]. However, in certain tumours this pathway is constitutively activated due to gain-of-function mutations in *NFE2L2* (encoding NRF2) or loss-of-function alterations in *KEAP1* or *CUL3*, promoting cell proliferation and drug resistance. NRF2 activation has been associated with poor response to several cancer therapies, including chemotherapy, radiotherapy, RTK inhibitors and ICIs [132–136]. NRF2 also plays a pivotal role in the metabolic reprogramming of cancer cells, redirecting glucose and glutamine utilisation toward anabolic pathways required to sustain proliferation. Among its effects, NRF2 upregulates the expression of enzymes involved in glutathione biosynthesis and inhibits ferroptosis, a form of programmed cell death driven by iron-dependent lipid peroxidation. By promoting glutamine uptake and increasing tumour cell dependence on external glutamine sources, NRF2 enhances sensitivity to glutaminase inhibitors, some of which are in clinical trials, albeit with limited results at present [137, 138].

Several NRF2 inhibitors, including small molecules, stapled peptides and natural products, have shown antitumor activity and synergism with other therapies in preclinical studies [139]. However, their mechanisms of action have not been confirmed in some cases, and no such compounds are currently in clinical development. Among new promising candidates are PROTACS [140] and the so-called molecular glues [141] such as R16, which binds specifically to a crevice in mutant KEAP1 and restores its binding affinity for NRF2 [142]. Targeting NRF2 epigenetic regulation and PTMs are other potential strategies [143]. In addition, NR0B1, an orphan nuclear receptor expressed in KEAP1-mutant NSCLC, has been identified as potential druggable target [144], while tumours with overactivation of NRF2 show selective vulnerability to inhibitors of respiratory complex I [145]. The TORC1/2 inhibitor TAK-228 has also shown promise in treating NSCLC with NRF2 pathway alterations [79], and combination therapies with glutaminase inhibitors may help overcome resistance in LUSC [146].

An additional tumour suppressor gene frequently mutated in NSCLC is STK11, which encodes the liver kinase B1 (LKB1) protein, a master kinase that regulates cell polarity, metabolism, proliferation and migration [147]. Unlike most kinases, which are activated by phosphorylation, LKB1 is allosterically activated through its interaction with the STE-20-related kinase adaptor protein (STRAD) and Mouse protein 25 (MO25), which together form a heterotrimeric complex with LKB1. LKB1 phosphorylates and activates AMP-activated protein kinase (AMPK). AMPK is also affected by the ratio of intracellular AMP to ATP, acting as an energy sensor, activated by AMP and deactivated by ATP. Activation of the LKB1–AMPK pathway stimulates catabolic processes and antagonises anabolic processes. It works, among other mechanisms, by inhibiting mTORC1, but also by inducing autophagy. Additionally, LKB1 activates a number of AMPK-related kinases through which it regulates pathways such as the Hippo pathway and EMT.

AMPK agonists are currently under investigation as potential anticancer therapies. The most extensively studied compound is metformin, which increases the cytoplasmic AMP: ATP ratio, thereby activating AMPK [148, 149]. However, the LKB1–AMPK pathway may also exert pro-tumorigenic effects, and its downregulation can enhance the efficacy of certain anticancer treatments. Therefore, the development of drugs targeting the LKB1–AMPK pathway requires a deeper understanding of the specific effects of its activation and inhibition across different cellular contexts [147].

Drugs targeting other tumour suppressor genes frequently altered in NSCLC, such as NF1 and SMARCA4, are being developed [150, 151].

## A roadmap to clinical implementation

The development of multi-omics assays faces several challenges related to the standardisation of omics techniques, data analysis and interpretation and clinical implementation.

Each omics technique must adhere to appropriate quality standards, from sample preparation to the technical execution of the assay and the data pre-processing required for analysis [16, 152–155]. Data formats differ significantly across omics platforms, ranging from qualitative to discrete or continuous quantitative data [156]. Analytical approaches span from classical statistical techniques to machine learning methods, although the boundary between these disciplines is often blurred.

Any omics data analysis must first deal with the 'curse of dimensionality,' whereby the high number of measured variables relative to the limited number of patients hinders parameter estimation in classical statistical models. This can dilute data correlations and complicate the identification of significant predictors, thereby reducing the performance of even machine learning models [157]. Additional issues include the need to correct for multiple testing [158] and the increased risk of overfitting as the number of analyzed variables grows [159].

As a result, dimensionality reduction techniques are often a necessary first step, using either linear or nonlinear methods [160, 161]. The choice between these techniques can significantly impact analytical outcomes and requires careful judgement. The study objective further dictates the choice between supervised analyses, aiming to identify differences between predefined categories of tumours or patients based on phenotype or outcome and unsupervised analyses, aiming to identify subgroups of patients or tumour subtypes based on biomolecular profile similarity within-subgroups and dissimilarity between-subgroups [159, 161].

There are various strategies for integrating data from different omics platforms [156, 161–164]. Early integration involves merging data from all omics types into a single large matrix and applying supervised or unsupervised analyses appropriate to the study objectives. Late integration involves analyzing each omics data type separately, then combining the results. Many approaches fall between these two extremes and are referred to as intermediate integration strategies.

Some analytical methods incorporate existing biological knowledge, such as functional genomics or functional proteomics, into omics data analysis, generally yielding results that are more accurate and biologically interpretable [165]. With different types of analyses, it is possible to reconstruct molecular networks involved in biological processes—such as gene regulatory networks, protein-protein interaction networks and others—and use them for data integration and interpretation [166]. Specific computational methods have been elaborated for single-cell transcriptomic and multiomic studies, including methods for spatial analysis [154, 155, 167].

Starting from a reduced set of variables, selected from multi-omics data using statistical and machine learning techniques, other analytical methods typical of systems biology can further characterise biological processes and potential interventions. These include, among others, logical models, aiming to capture the qualitative behaviour of biological systems and the causal influences between variables and models based on systems of differential equations, including stochastic ones, attempting to quantitatively describe the mechanisms underlying biological processes [168, 169]. These models require in-depth biological knowledge but can offer insightful interpretations and accurate predictions, potentially enabling the development of patient-specific models testable in biological systems such as organoids or patient-derived xenografts [170, 171]. The use of these models might require measuring only a limited number of biomolecular variables in individual patients, selected based on tumour type, through a form of targeted multi-omics analysis.

Clinical application of predictive multi-omics models requires evidence of clinical utility, which must follow the established criteria for developing biomarkers [172, 173]. These include demonstrating analytical validity (accuracy, reproducibility, reliability), clinical validity (ability to identify distinct patient subgroups based on biology or outcomes) and clinical utility, i.e., evidence that using the biomarker to guide therapy improves patients’ outcomes, or maintains similar outcomes with reduced toxicity and/or costs, compared to standard clinic-pathological criteria. The highest level of evidence comes from randomised clinical trials specifically designed to demonstrate the biomarker’s clinical utility in the intended setting.

Various clinical trial designs have been developed for this purpose, including adaptive trials that dynamically identify the patients’ subset benefiting from the tested treatment [174]. While prospective demonstration of the utility of transcriptomic predictors in breast cancer required large and long-duration trials [175, 176], increasingly accurate predictors may significantly reduce the number of patients needed for clinical validation. Moreover, as each tumour is biologically unique, n-of-1 trials may be appropriate [177–179], wherein each patient receives the treatment (mono- or poly-pharmacologic) deemed optimal for their specific tumour. Meta-analyses of individualised therapy outcomes from multiple n-of-1 trials can then be compared with standard treatment results. Additional opportunities may help optimise clinical research. Mathematical models that simulate tumour growth and its reduction in response to treatment, calibrated with individual patient data, can serve as patients’ digital twins. These models have the potential to enable personalised clinical trials by simulating tumour evolution in response to various treatment options, thereby supporting the identification of the most effective therapy and allowing for the adjustment of specific treatment parameters [180]. They can be integrated with data-driven models, generating predictions through machine learning algorithms [181], as well as with biology-based models that describe biological mechanisms through systems of differential equations.

## Conclusions

Targeted genomic profiling is a standard in the diagnostic workup of NSCLC and essential for guiding treatment in patients with actionable mutations. This approach has transformed the management of NSCLC and laid the groundwork for precision oncology. However, for tumours lacking actionable alterations, treatment relies primarily on ICIs with or without chemotherapy, despite suboptimal predictive biomarkers and heterogeneous response rates.

Genomic information alone is often insufficient to identify optimal therapeutic targets. The integration of diverse omics technologies—genomics, transcriptomics, proteomics, epigenomics and others—offers a more comprehensive view of tumour biology. Proteomics, in particular, provides insight into cellular phenotypes and the activity of intracellular signalling networks through protein-level information and post-translational modifications. These integrated approaches enable tumour subtyping, elucidate signalling derangements and can support the identification of therapeutic targets [17, 21].

As described throughout this review, NSCLCs lacking currently actionable mutations exhibit a complex molecular landscape. This includes rare but targetable alterations (Tables 1–3), epigenetic modifications under investigation and frequent mutations in tumour suppressor genes that remain non-targetable yet are intensively studied. The strength of the multi-omics approach lies in its ability to reveal mechanistic links across molecular layers. For instance, BZW2, which has limited prognostic relevance [182], was found to be upregulated in proteogenomic analyses [69] and later shown to promote LUAD progression and represent a potential therapeutic target [183–185]. The observed discordance between mRNA and protein levels has helped clarify the effects of some specific KEAP1 mutations on NRF2 activation in LUAD [69]. In LUSC, activation of the NRF2 pathway was identified even in the absence of direct mutations within the pathway itself [103], broadening the spectrum of potential use of NRF2 pathway inhibitors.

Single-cell and spatial multi-omics analyses further dissect tumour and stromal cell interactions, highlighting predictors of response to ICIs [113] and supporting their use in this and other future applications.

Despite their promise, multi-omics approaches remain primarily investigational. Their translation to clinical practice requires technical standardisation, selection of relevant variables and rigorous clinical validation. Some molecular layers, such as noncoding RNAs, epigenetics and metabolomics, are still underexplored and need better integration.

Several NSCLC alterations affect non-actionable genes, including tumour suppressors and oncogenes like MYC [186]. Research into targeting these pathways remains critical to expanding therapeutic options.

In conclusion, genomic profiling remains central to both clinical decision-making and research. While many alterations may not yet inform treatment choices, they are essential for discovering new targets. Multi-omics strategies can further deepen our understanding of NSCLC biology, with the potential to reveal novel therapeutic targets and resistance mechanisms, thereby supporting precision oncology. With rigorous validation, multi-omics analysis can pave the way for new advances in personalised cancer care.
